# LncRNA MALAT1 Promotes PPARα/CD36-Mediated Hepatic Lipogenesis in Nonalcoholic Fatty Liver Disease by Modulating miR-206/ARNT Axis

**DOI:** 10.3389/fbioe.2022.858558

**Published:** 2022-06-13

**Authors:** Juan Xiang, Yuan-Yuan Deng, Hui-Xia Liu, Ying Pu

**Affiliations:** ^1^ Endocrinology Subspecialty of Geriatrics, Xiangya Hospital of Central South University, Changsha, China; ^2^ National Clinical Research Center for Geriatric Disorders, Xiangya Hospital of Central South University, Changsha, China

**Keywords:** non alcoholic fatty liver disease, lncRNA MALAT1, hepatic lipogenesis, miR-206, ARNT, PPARα/CD36

## Abstract

Long non-coding RNAs (lncRNAs) are known to play crucial roles in nonalcoholic fatty liver disease (NAFLD). This research sought to explore mechanisms by which lncRNA MALAT1 regulates the progression of NAFLD. Thus, in order to detect the function of MALAT1 in NAFLD, *in vitro* and *in vivo* model of NAFLD were established. Then, fatty acid uptake and triglyceride level were investigated by BODIPY labeled-fatty acid uptake assay and Oil red O staining, respectively. The expressions of MALAT1, miR-206, ARNT, PPARα and CD36 were detected by western blotting and qPCR. Dual luciferase, RIP and ChIP assay were used to validate the relation among MALAT1, miR-206, ARNT and PPARα. The data revealed expression of MALAT1 was up-regulated *in vitro* and *in vivo* in NAFLD, and knockdown of MALAT1 suppressed FFA-induced lipid accumulation in hepatocytes. Meanwhile, MALAT1 upregulated the expression of ARNT through binding with miR-206. Moreover, miR-206 inhibitor reversed MALAT1 knockdown effects in decreased lipid accumulation in FFA-treated hepatocytes. Furthermore, ARNT could inhibit the expression of PPARα via binding with PPARα promoter. Knockdown of MALAT1 significantly upregulated the level of PPARα and downregulated the expression of CD36, while PPARα knockdown reversed these phenomena. MALAT1 regulated PPARα/CD36 -mediated hepatic lipid accumulation in NAFLD through regulation of miR-206/ARNT axis. Thus, MALAT1/miR-206/ARNT might serve as a therapeutic target against NAFLD.

## Introduction

Nonalcoholic fatty liver disease (NAFLD) is characterized by excessive accumulation of intracellular fat which was not caused by alcohol and other specific factors, and it can lead to metabolic stress related with genetic susceptibility (Loomba and Sanyal, 2013; Byrne and Targher, 2015). The incidence rate of NAFLD is 10–30% among which 10–20% are nonalcoholic steatohepatitis, and the incidence of cirrhosis is 25% in 10 years (Kim et al., 2019). The progression of NAFLD can affect the life quality of patients. Thus, exploring the pathogenesis of NAFLD and finding the effective therapies against NAFLD are highly desirable.

Long non-coding RNA (lncRNA) (more than 200 nucleotides) is involved in the progression of NAFLD (Jathar et al., 2017; Robinson et al., 2020). For example, lncRNA NEAT1 was up-regulated in NAFLD and knockdown of NEAT1 could alleviate the development of NAFLD (Ye et al., 2020); lncRNA H19 was positively correlated with hepatic lipogenesis in NAFLD (Wang et al., 2020). Some studies explored the role of metastasis-associated lung adenocarcinoma transcript 1 (MALAT1) in cancer (Li et al., 2015). Besides, MALAT1 was upregulated in patients with NAFLD, contributing to the progression of liver fibrosis (Sookoian et al., 2018). However, the role of MALAT1 in liver fat metabolism remains unclear. MiR-206 was reported to inhibit fat synthesis in liver (Yu et al., 2015). In addition, the expression of ARNT was highly associated with obesity (Wu et al., 2017), and it was downregulated in NAFLD (Paschos et al., 2012). According to previous research results (Mouralidarane et al., 2015) and our bioinformatics predicted results, we suspected there were binding site between miR-206 and MALAT1 or aryl hydrocarbon receptor nuclear translocator (ARNT). Nevertheless, the relationship among MALAT1, miR-206 and ARNT in NAFLD remains unknown.

Peroxisome proliferator activated receptor-α (PPARα) and fatty acid transporter (CD36) participates in the NAFLD process, and CD36 also serves as an important target for PPARα according to the previous reports (Chen et al., 2019). PPARα could be activated by intracellular fatty acids (FAs) such as docosahexaenoic acid (DHA) to accelerate fatty acid oxidation and gluconeogenesis. In addition, CD36 is relatively down-regulated in hepatocytes (Mou et al., 2021). Mice with high fat diet could result in an increase of CD36 level, leading to fatty liver (He et al., 2011). Clinically, higher level of CD36 was closely correlated with NAFLD, and this phenomenon was accompanied by production of hepatic fatty acids (Greco et al., 2008). It was reported that CD36 was associated with pregnane X receptor (PXR), PPARs and the liver X receptor (LXRα) (Li et al., 2019). However, the detailed association between MALAT1 and PPARα/CD36 pathway in NAFLD requires further exploration.

Based on the above backgrounds, we hypothesized MALAT1 could regulate PPARα/CD36 through mediation of miR-206/ARNT axis, therefore modulating the synthesis of hepatocyte fat. This study might shed new lights on exploring the theoretical basis and targets against NAFLD.

## Materials and Methods

### Ethics Statements

The procedures of the animal experiments were approved by the Ethics Committee of Xiangya Hospital of Central South University and followed the guidelines for the Use and Care of Laboratory Animals by International Committees.

### Sample Collection

Normal liver tissues (*n* = 10) and NAFLD liver tissues (*n* = 20) were obtained from Xiangya Hospital of Central South University between August 2019 and September 2020. Liver tissues in NAFLD were obtained from patients with NAFLD. Normal liver tissues were obtained from patients who had been in a car accident. Clinical samples were collected: the participants were aged 18–64 years old and had a body mass index (BMI) ≥ 25 kg/m^2^; The following were the exclusion criteria: Individuals who reported ongoing or recent alcohol consumption (men’s standard drinks 21/week and women’s standard drinks 14/week); people who had a history of exposure to hepatotoxic drugs, with liver disease or liver cancer; had an active chronic gastrointestinal disorder (e.g., inflammatory bowel disease, ulcerative colitis, celiac disease) or taking any medication or supplement known to affect body composition (Wilson et al., 2016). The present study was approved by the Institutional Ethical Committee of Xiangya Hospital of Central South University (IRB{S}NO. 202104073). Written informed consent was obtained from all participants.

### Immunohistochemical Staining

Liver tissues of patients were fixed in 4% paraformaldehyde in PBS overnight, paraffin-embedded, and cut into 5-μm-thick sections. Paraffin sections were deparaffinized and rehydrated. The sections were heated in sodium citrate buffer in a microwave for antigen retrieval, washed with phosphate-buffered saline (PBS) for 5 min (three times) at room temperature, incubated in 3% H_2_O_2_ at room temperature for 25 min, washed with PBS for 5 min (three times) and blocked and incubated in goat serum for 30 min. Then, the samples were stained with primary antibodies (anti-ARNT, anti-CD36 and anti-PPARα) overnight at 4°C. After that, samples were incubated with secondary antibody (HRP-labeled) for 30 min at 37°C. Finally, freshly prepared diaminobenzidine (DAB) was added for colour development. All the antibodies were obtained from Abcam. The tissues were observed under a fluorescence microscope.

### NAFLD Animal Model

The NAFLD animal model was in line with a previously report (Zhou et al., 2008). Male C57BL/6 mice (8 weeks old, *n* = 6/group) were from Vital River Laboratory Animal Technology (Beijing, China). Four groups included standard chow diet (SCD; 65% carbohydrate, 10% fat and 25% protein) group, high fat diet (HFD; 35% carbohydrate, 50% fat and 15% protein) group, HFD + sh-NC group and HFD + sh-MALAT1 group. After 12 weeks of HFD treatment, mice were sacrificed for collection of blood samples and liver tissues. The standard feed and high fat feed used in the study were customized by Beijing Keao Xieli Feed Co., Ltd (Beijing, China).

### Cell Cultivation and Treatment

HepG2 cells were obtained from the American Type Culture Collection (ATCC, Manassas, VA, United States). The culture condition was Dulbecco’s modified Eagle’s medium (DMEM; Gibico, United States) containing 10% fetal bovine serum (FBS; Invitrogen, Carlsbad, CA, United States) in 5% CO_2_ and 37°C. To mimic NAFLD *in vitro*, HepG2 cells were treated with 1 mM free fatty acids (FFA; oleate: palmitate = 2:1) for 24 h. Subsequently, cells in each group were isolated for RNA/protein, and then the isolated proteins were used for further study.

### Cell Transfections

The shRNA MALAT1 (sh-MALAT1), sh-negative control (sh-NC), miR-206 inhibitor, inhibitor NC, miR-206 mimics, miR-NC, sh-ARNT and sh-PPARα were obtained from GenePharma (Shanghai, China). HepG2 cells transfected with sh-MALAT1, sh-NC, miR-206 inhibitor, inhibitor NC, miR-206 mimics, miR-NC, sh-ARNT or sh-PPARα by using Lipofectamine 3000 (Invitrogen).

### Hematoxylin and Eosin Staining

The liver tissues of mice were extracted and then fixed with 4% polyformaldehyde in phosphate buffer, after which the samples were dehydrated and embedded with paraffin. Subsequently, the as-prepared paraffin block was sliced into 4 μm slices, and then stained with 5% hematoxylin (10 min) and 1% eosin (5 min). Finally, the samples were observed with a microscopy.

### Oil Red O Staining

First, the treated frozen sections of liver tissues and HepG2 cells were subjected to 4% paraformaldehyde (10 min). After washing, Oil-Red O staining solution was added in both liver samples and cell samples for 10 min and hematoxylin staining solution was used for nuclear staining. Results of lipid accumulation were inspected under microscope (Carl Zeiss MicroImaging GmbH, Göttingen, Germany).

### Triglyceride Assay

A TG assay kit (Applygen Technologies Inc., Beijing, China) was used to examine TG level in lysed cells. TG concentration was normalized to total protein concentration which was detected via the BCA method, expressing as mmol/mg protein.

### Quantitative Real-Time PCR

The total RNA of transfected cells and liver tissues were first collected with a TRIzol reagent (Invitrogen). After that, the reverse transcriptions of RNA into cDNA were conducted through a reverse transcriptase kit from Invitrogen Company. The used primers were shown in Table 1. PCR processes were conducted on ABI 7500 Real-Time PCR System. The relative expression levels of RNA normalized to GAPDH or U6 were calculated using the 2^−∆∆Ct^ method.

**TABLE 1:** Primers used for qPCR assay.

Gene	Sequence (5’→ 3′)
MALAT1 (human)	Forward: AAA​GCA​AGG​TCT​CCC​CAC​AAG
	Reverse: GGT​CTG​TGC​TAG​ATC​AAA​AGG​CA
MALAT1 (mouse)	Forward: GGG​GGA​ATG​GGG​GCA​AAA​TA
	Reverse: AAC​TAC​CAG​CAA​TTC​CGC​CA
PPARα (human)	Forward: GAC​AAG​GCC​TCA​GGC​TAT​CA
	Reverse: GTT​GTG​TGA​CAT​CCC​GAC​AG
PPARα (mouse)	Forward: AGT​GCA​GCC​TCA​GCC​AAG​TT
	Reverse: CAC​AGA​GCG​CTA​AGC​TGT​GA
CD36 (human)	Forward: GCC​AGT​TGG​AGA​CCT​GCT​TA
	Reverse: CTC​AGC​GTC​CTG​GGT​TAC​AT
CD36 (mouse)	Forward: GTG​TGG​AGC​AAC​TGG​TGG​AT
	Reverse: ACG​TGG​CCC​GGT​TCT​AAT​TC
SREBP1 (human)	Forward: GGA​GCC​ATG​GAT​TGC​ACT​TTC​G
	Reverse: GCT​CAG​GAA​GGC​TTC​AAG​AGA​G
ACC1 (human)	Forward: CAA​GGT​CAG​CTG​GTC​CAC​ATG
	Reverse: GTG​GAA​TAC​CTT​CTG​CCC​TAG​C
SCD1 (human)	Forward: CTT​GCG​ATA​TGC​TGT​GGT​GC
	Reverse: AAG​TTG​ATG​TGC​CAG​CGG​TA
FASN (human)	Forward: TAT​GAA​GCC​ATC​GTG​GAC​GG
	Reverse: GAA​GAA​GGA​GAG​CCG​GTT​GG
miR-206 (human)	Forward: GGC​GGT​GGA​ATG​TAA​GGA​AG
	Reverse: CGG​CCC​AGT​GTT​CAG​ACT​AC
miR-206 (mouse)	Forward: GCC​GAG​TGG​AAT​GTA​AGG​AAG​T
	Reverse: GTC​GTA​TCC​AGT​GCA​GGG​TCC​GAG​G
	TAT​TCG​CAC​TGG​ATA​CGA​CCC​ACA​C
ARNT (human)	Forward: CAA​GCC​CCT​TGA​GAA​GTC​AG
	Reverse: GGG​GTA​GGA​GGG​AAT​GTG​TT
ARNT (mouse)	Forward: CCC​AGG​CTA​CAG​CCA​AGA​C
	Reverse: CCT​GGA​ACT​GTC​CTG​TGG​TC
U6	Forward: CTCGCTTCGGCAGCACA
	Reverse: AAC​GCT​TCA​CGA​ATT​TGC​GT
GAPDH	Forward: AGG​TCG​GAG​TCA​ACG​GAT​TT
	Reverse: TGA​CGG​TGC​CAT​GGA​ATT​TG
GAPDH (mouse)	Forward: AGC​CCA​AGA​TGC​CCT​TCA​GT
	Reverse: CCG​TGT​TCC​TAC​CCC​CAA​TG

GAPDH, denoted as glyceraldehyde-3-phosphate dehydrogenase.

### Western Blotting Analysis

Total protein from liver tissues or cells was isolated by using lysis buffer. Then, extracted proteins were first transferred into the buffer solution, then boiled for 5 min at 100°C, and collected with centrifugation. Subsequently, 30 µg protein samples were separated by SDS-PAGE and then transferred to PVDF membrane (Millipore, Merck Shanghai, United States). After incubating with 5% skim milk, the membrane was placed with primary antibodies (anti-PPARα, ab126285; anti-CD36, ab252922; anti-ACC1, ab109368; anti-SCD1, ab236868 at 1:1000, from Abcam; anti-ARNT, PA5-106857 at 1:1000, from Sigma; anti-SREBP1, NB100-2215; anti-FASN, AF5927 at 1:1000, from Novus). After washing, membrane was incubated with horseradish peroxidase (HRP)-conjugated secondary antibody (1:3000; Abcam) for 1 h. Lastly, enhanced chemiluminescence system (ECL; Millipore) was used to visualize protein bands and relative densitometry was analyzed using ImageJ software.

### BODIPY-Labeled Fatty Acid Uptake Assay

BODIPY fatty acid uptake experiment was referred to Khalifeh-Soltani et al. (Garcia et al., 2022). In brief, 1 µM BODIPY-C16 (Invitrogen) was allowed for incubation with HepG2 cells at 37°C for 30 min. Then, surface-associated BODIPY was removed at 4°C with 0.2% BSA in PBS. Finally, the fluorescence of BODIPY was detected via a fluorescence plate reader.

### Biochemical Analysis

Mouse blood samples were obtained by eye-balls removal method. Using a biochemical auto-analyzer (Fuji Medical System, Tokyo, Japan), total cholesterol (TC), TG, alanine aminotransferase (ALT) and aspartate aminotransferase (AST) levels were detected.

### Dual-Luciferase Reporter Assay

The wild-type/mutated 3′UTRs of ARNT (ARNT-WT/ARNT-MUT) luciferase vectors were formed by using amplified DNA sequences cloned into pmirGLO reporter plasmids (Promega, Madison, WI, United States). And the MALAT1-WT/MALAT1-MUT luciferase reporter vectors constructed as above. HepG2 cells were co-transfected with luciferase vectors and miR-206 mimics or miR-NC with Lipofectamine 3000 transfection reagent (Invitrogen). The luciferase activities were measured after 48 h transfection. The relationship between PPARα promoter and ARNT was also ascertained by luciferase assay as described above.

### RNA Immunoprecipitation Assay

The RIP assay was conducted by adopted an EZ-Magna RIP kit (Millipore). First, lysis buffer was used to lyse the collected cells, and then magnetic beads with antibody targeting Ago2 or IgG as well as RIP buffer were added into the lysate for incubation overnight at 4 °C. After that, proteinase K was employed to incubate with these magnetic beads, and total RNAs were subsequently isolated for the relative enrichment of MALAT1 and miR-206 analysis via qPCR.

### Chromatin Immunoprecipitation Assay

HepG2 cells at the density of 2 × 10^6^ cells/mL were used for this experiment. After adding formaldehyde, the cell suspension was rotated gently for 10 min. Then glycine was incubated for 5 min crosslink. After centrifuging at 1000 × g for 5 min, the pellet was resuspended in nuclear lysis buffer containing protease and phosphatase inhibitor cocktail. The sheared chromatin was obtained using a probe sonicator and proceeded to immunoprecipitation. In brief, primary antibody was added and sample was rotated overnight and Dynabeads was incubated for 2 h. After elution and purification, sample was proceeded with ChIP-qPCR.

### Statistical Analysis and Bioinformatics Prediction

All data were presented as mean ± SD and GraphPad Prism 6 software was used for statistical analysis. Student’s *t* test was used to assess the difference between two groups. One-way ANOVA was adopted when three or more groups were compared. *p* value <0.05 represented the significant difference. StarBase (http://starbase.sysu.edu.cn/) and miRmap (https://www.mirmap.ezlab.org/app/) were applied to predict the binding sites between miR-206 and MALAT1/ARNT. The interaction of ARNT and PPARα promoter was predicted via JASPAR (http://jaspar.genereg.net/). Meanwhile, three independent experiments were performed in this research.

## Results

### MALAT1 was Upregulated in NAFLD

**FIGURE 1:**
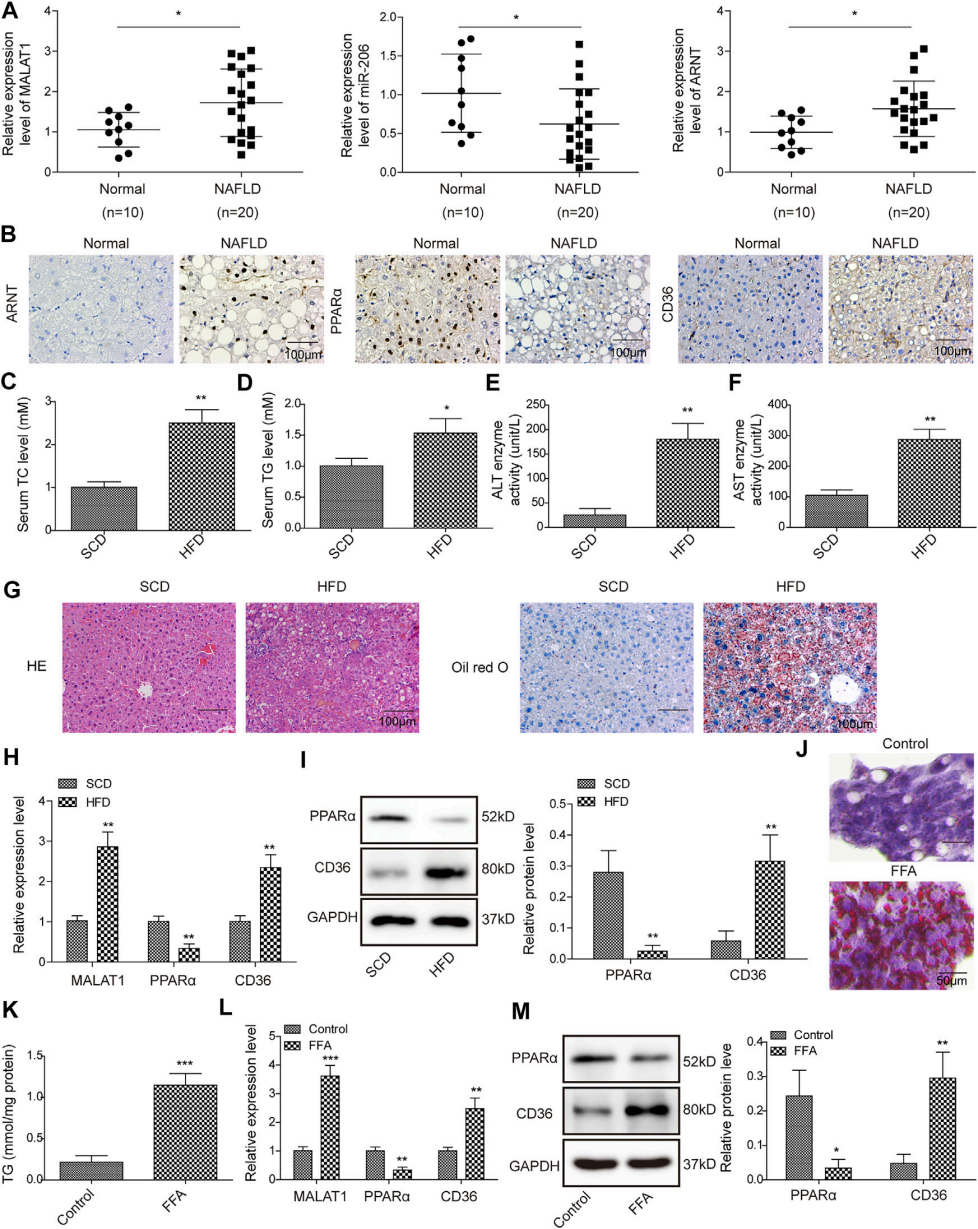
MALAT1 was up-regulated in NAFLD. **(A)** The levels of MALAT1, miR-206 and ARNT in normal liver tissues or NAFLD liver tissues were detected by qPCR. **(B)** The levels of ARNT, PPARα and CD36 in liver tissues were detected by IHC staining. Normal (*n* = 10), NAFLD (*n* = 20) **(C–F)** TC, TG levels, AST and ALT enzyme activities in serum of NAFLD mice were detected with automatic biochemical analyzer. **(G)** Hepatic steatosis was assessed by H&E and Oil Red O staining. **(H)** Relative RNA levels of MALAT1, PPARα and CD36 in liver tissues were measured by qPCR assay. **(I)** The protein levels of PPARα and CD36 were determined by western blotting assay. *n* = 6. **(J)** Oil Red O staining was used for lipid accumulation detection. **(K)** TG level was detected by the TG assay kit. **(L)** MALAT1, PPARα and CD36 levels in FFA-induced HepG2 cells were determined by qPCR. **(M)** PPARα and CD36 levels in FFA-treated cells were determined by western blotting. Data were presented as the mean ± SD. Student’s *t*-test was used to assess the difference between two groups. One-way analysis of variance was used among multiple groups. *n* = 3, *p < 0.05, **p < 0.01.

In order to investigate the role of MALAT1, miR-206 and ARNT in NAFLD, qPCR was performed. As revealed in Figure 1A, the levels of MALAT1 and ARNT in NAFLD tissues were significantly higher, compared with those in normal tissues. In contrast, the level of miR-206 in NAFLD tissues was much lower than that in normal tissues (Figure 1A). In addition, the data of IHC showed that the levels of ARNT and CD36 in NAFLD tissues were significantly up-regulated, while the levels of PPARα were down-regulated (Figure 1B). *In vivo* model of NAFLD was firstly established, and then the following experiments were carried out. As shown in Figures 1C–F, the levels of TC, TG and the enzyme activities of ALT and AST in serum of mice were up-regulated in the HFD group than those in SCD group. In addition, obvious steatosis and excessive accumulation of lipids were observed in HFD mice (Figure 1G). The levels of MALAT1 and CD36 were markedly increased, while the expression of PPARα was apparently downregulated in HFD mice (Figures 1H,I). Besides, FFA treatment remarkably promoted lipid accumulation and increased the level of TG in HepG2 cells (Figures 1J,K). Meanwhile, the expressions of MALAT1 and CD36 in HepG2 cells were up-regulated by FFA, while the level of PPARα was downregulated (Figures 1L,M). Together, MALAT1 was up-regulated in NAFLD.

### Knockdown of MALAT1 Reversed FFA-Induced Lipid Accumulation in HepG2 Cells

**FIGURE 2:**
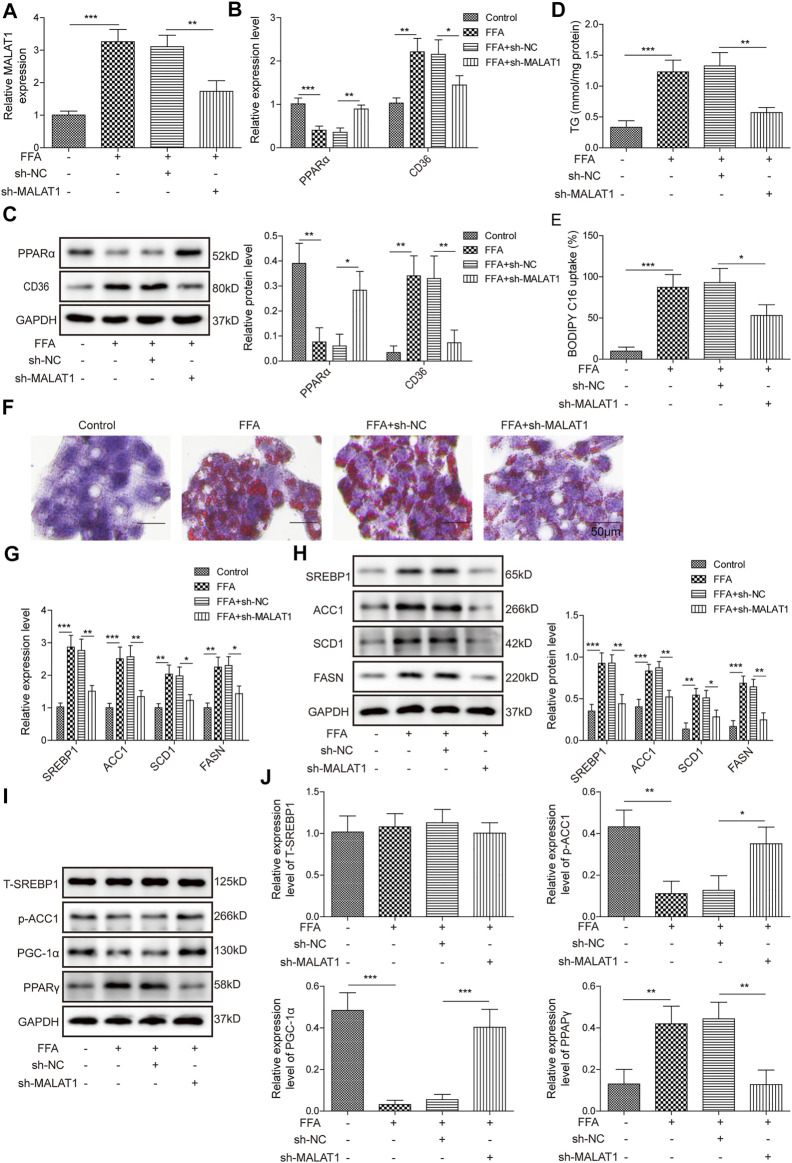
Knockdown of MALAT1 reversed FFA-induced lipid accumulation. **(A)** The expression of MALAT1 was detected by qPCR. **(B,C)** Expression of PPARα and CD36 were determined by qPCR and western blotting assays. **(D)** The level of TG in HepG2 cells was detected by biochemical kits. **(E)** The BODIPY C16 uptake in HepG2 cells after transfection with sh-MALAT1. **(F)** Lipid accumulation in sh-MALAT1 transfected cells was assessed by Oil Red O staining. **(G,H)** Expression of SREBP1, ACC1, SCD1, and FASN determined by qPCR and western blotting assays. **(I,J)** The protein expressions of T-SREBP1, p-ACC1, PGC-1α and PPARγ in cells were detected by western blotting. Data were presented as the mean ± SD. One-way analysis of variance was used among multiple groups. *n* = 3, *p < 0.05, **p < 0.01.

The level of MALAT1 was increased by FFA in HepG2 cells, while sh-MALAT1 dramatically reversed FFA-induced increase of MALAT1 expression (Figure 2A). Additionally, knockdown of MALAT1 facilitated the expression of PPARα and downregulated the expression of CD36 (Figures 2B,C). As expected, FFA-induced increase of TG level was restored in the presence of MALAT1 knockdown (Figure 2D). Knockdown of MALAT1 could suppress FFA uptake and lipid accumulation in FFA-treated hepatocytes (Figures 2E,F). It is worth mentioning that FFA-increased lipid synthesis-related proteins (SREBP1, ACC1, SCD1, FASN) were markedly abolished by MALAT1 silencing (Figures 2G,H). Besides, FFA-induced decrease of p-ACC1 and PGC-1α levels was significantly rescued by knockdown of MALAT1, and MALAT1 shRNA notably restored the effect of FFA on the level of PPARγ (Figures 2I,J). These results demonstrated that knockdown of MALAT1 could reverse FFA-induced lipid accumulation via mediation of PPARα/CD36 axis.

### LncRNA MALAT1 Promotes the Expression of ARNT Through Binding With miR-206

**FIGURE 3:**
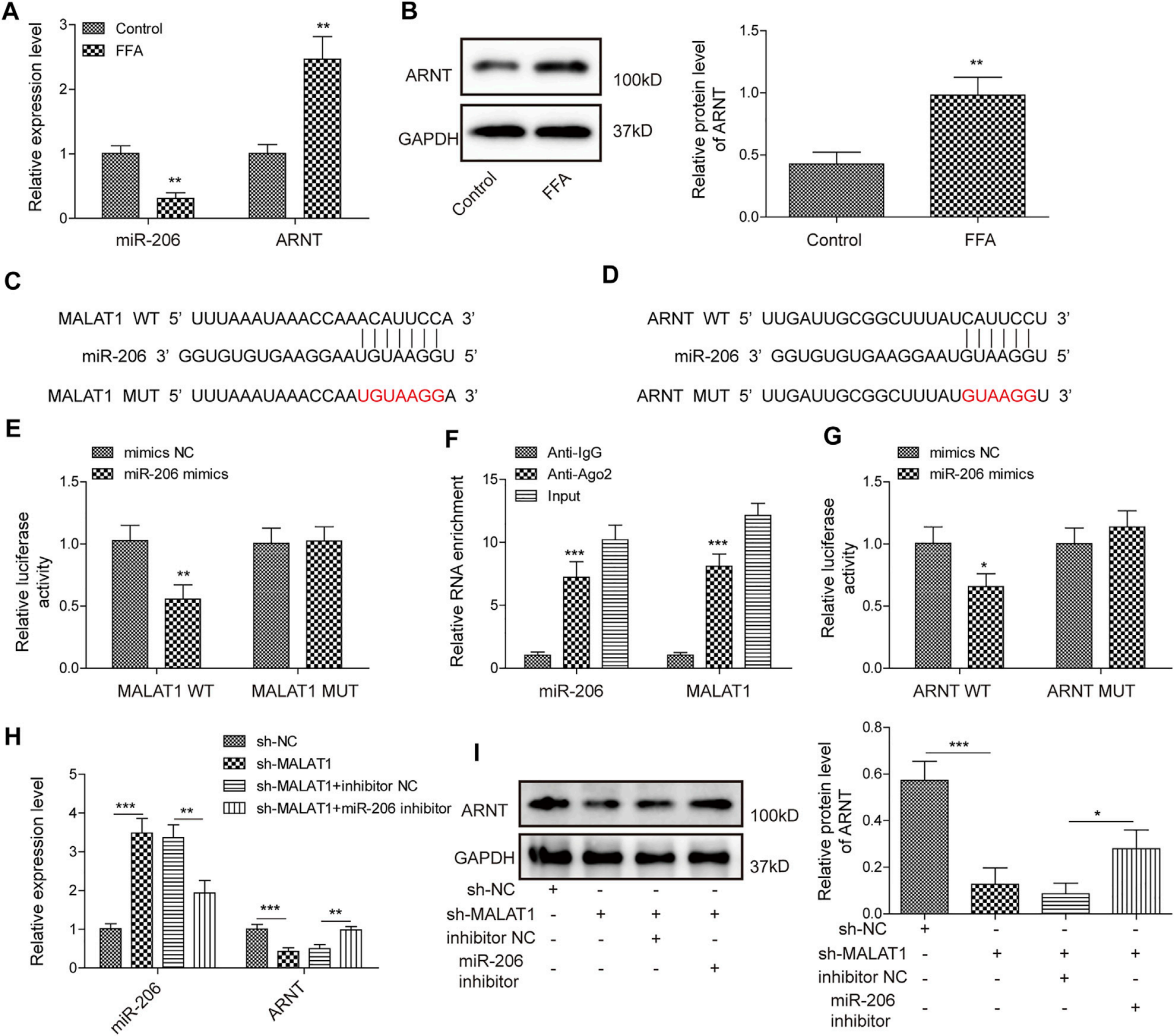
LncRNA MALAT1 could promote the expression of ARNT through sponging miR-206. **(A)** Expression levels of miR-206 and ARNT were detected by qPCR. **(B)** ARNT expression was determined by western blotting analysis. **(C,D)** Bioinformatics prediction of the binding sites between miR-206 and MALAT1/ARNT. **(E,F)** RIP and luciferase assays were used for determining the relationship between MALAT1 and miR-206. **(G)** Luciferase assays were used for measurement of relationship between miR-206 and ARNT. **(H,I)** Expression of miR-206 and ARNT in HepG2 cells transfected with sh-MALAT1 or/and miR-206 inhibitor. Data were presented as the mean ± SD. Student’s *t*-test was used to assess the difference between two groups. One-way analysis of variance was used among multiple groups. *n* = 3, *p < 0.05, **p < 0.01.

Next, we further investigated the mechanisms by which MALAT1 regulates lipid accumulation in hepatocytes. As shown in Figures 3A,B, FFA markedly downregulated the level of miR-206, while increased the expression of ARNT. Furthermore, miR-206 had binding sites with MALAT1/ARNT (Figures 3C,D). Moreover, miR-206 overexpression notably decreased the luciferase activity in MALAT1-WT reporter, while no obvious changes was found in MALAT1-MUT reporter group (Figure 3E). In addition, RIP analysis showed that MALAT1 and miR-206 were notably enriched in Ago2 (Figure 3F), and miR-206 distinctly decreased the luciferase activity of ARNT-WT reporter (Figure 3G). Knockdown of MALAT1 evidently upregulated the level of miR-206 and downregulated the level of ARNT; more importantly, miR-206 downregulation partially reversed the effects of sh-MALAT1 (Figures 3H,I). Therefore, MALAT1 upregulated the expression of ARNT through sponging miR-206.

### MiR-206 Down-Regulation Reversed the Effects of MALAT1 Knockdown on FFA-Induced Lipid Accumulation

**FIGURE 4:**
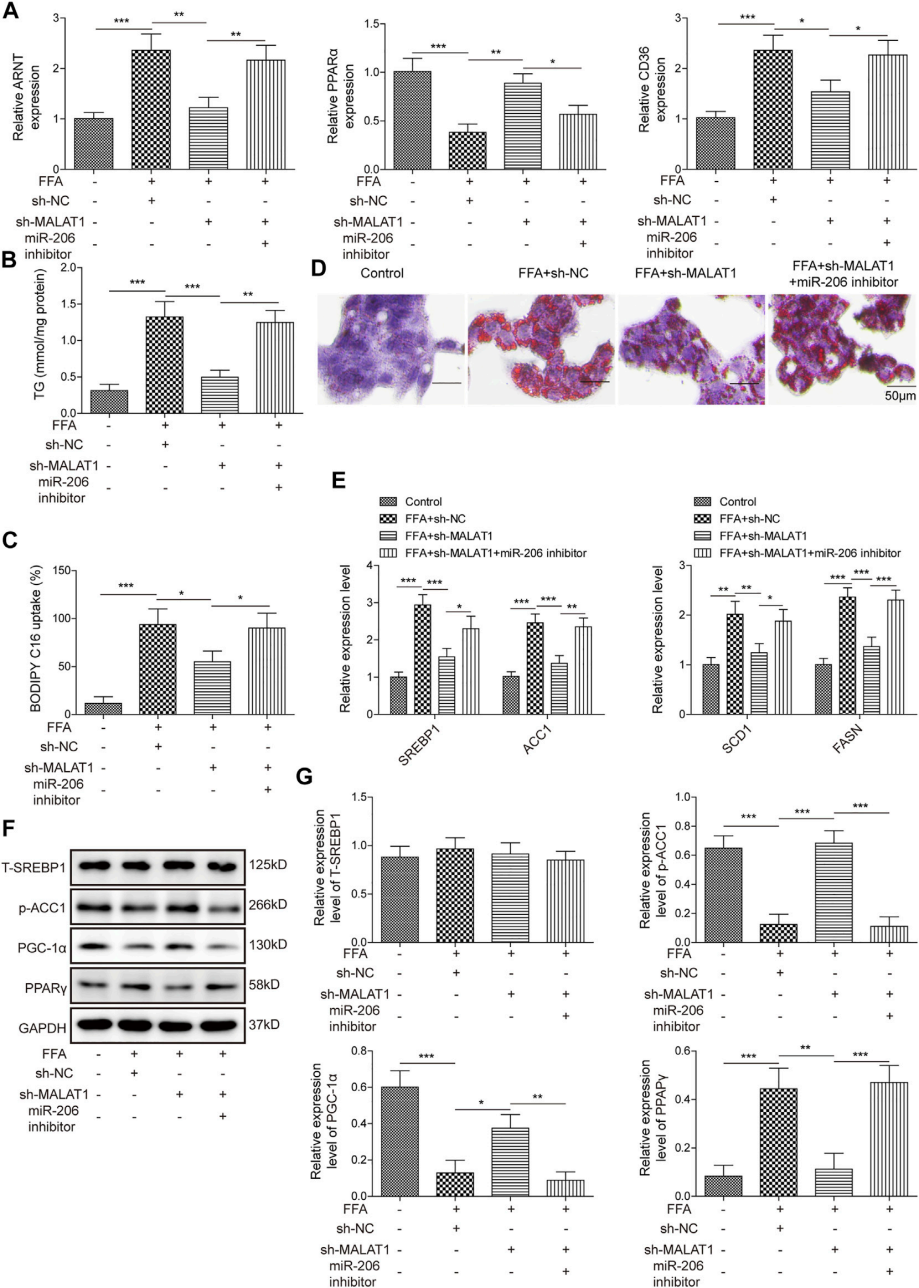
Inhibition of miR-206 reverses the effects of MALAT1-knockdown on lipid accumulation induced by FFA. **(A)** Expression levels of ARNT, PPARα and CD36 detected by qPCR. **(B)** TG levels detected by the TG assay kit. **(C)** Bodipy-labeled fatty acid uptake assay was used for assessment of fatty acid uptake. **(D)** Lipid accumulation was detected by Oil Red O staining. **(E)** Expression levels of SREBP1, ACC1, SCD1 and FASN detected by qPCR. **(F,G)** The protein expressions of T-SREBP1, p-ACC1, PGC-1α and PPARγ in cells were detected by western blot. Data were presented as the mean ± SD. One-way analysis of variance was used among multiple groups. *n* = 3, *p < 0.05, **p < 0.01, ***p < 0.001.

To further ascertain the regulatory function of MALAT1, we then investigated the effects of miR-206 on lipid accumulation in hepatocytes. As shown in Figure 4A, MALAT1 knockdown could downregulate the expression of ARNT, CD36 and upregulate the expression of PPARα, while the inhibition of miR-206 partially reversed the effects of sh-MALAT1. In addition, miR-206 down-regulation also reversed the effects of MALAT1 silencing on TG level in FFA-treated HepG2 cells (Figure 4B). Furthermore, from BODIPY-labeled fatty acid uptake assay (Figure 4C), the effects of sh-MALAT1 on FFA uptake were partially decreased with the inhibition of miR-206. Via Oil Red O staining, the same trends can be found in lipid accumulation (Figure 4D). Moreover, miR-206 downregulation significantly up-regulated the expression of SREBP1, ACC1, SCD1 and FASN, while MALAT1 knockdown exhibited the opposite (Figure 4E). Furthermore, the effect of MALAT1 shRNA on p-ACC1, PPARγ and PGC-1α levels in FFA-induced cells was significantly reversed by miR-206 inhibitor (Figures 4F,G). All these results proved that miR-206 inhibitor reversed the effect of MALAT1 silencing on FFA-induced lipid accumulation.

### ARNT Regulates the Transcription of PPARα Through Binding to PPARα Promoter

**FIGURE 5:**
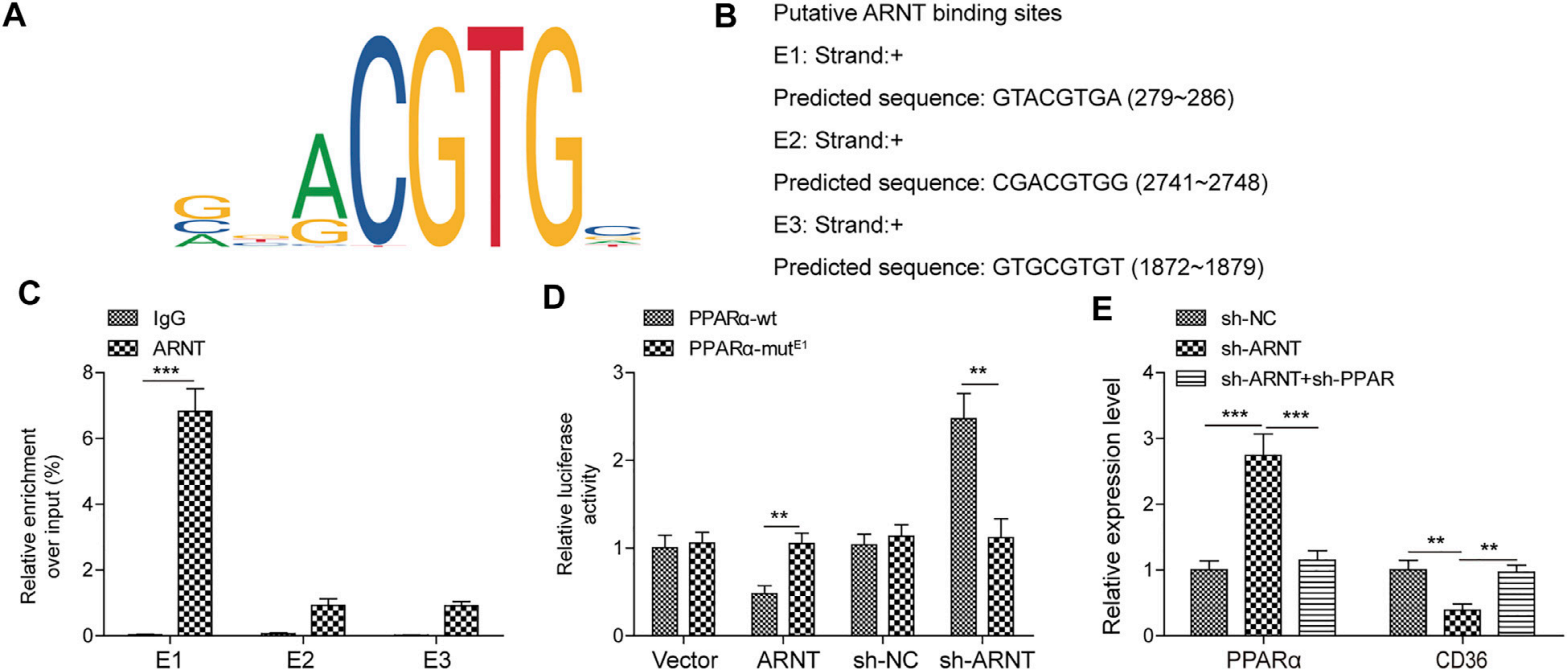
ARNT binds to the PPARα promoter and regulates its transcription. **(A)** Binding Motif of ARNT (from JASPAR). **(B)** JASPAR predicts the first three sites at which ARNT binds to PPARα promoters. **(C,D)** ChIP/Luciferase validates that ARNT binds to the PPARα promoter. **(E)** PPARα and CD36 levels were detected by qPCR. Data were presented as the mean ± SD. Student’s *t*-test was used to assess the difference between two groups. One-way analysis of variance was used among multiple groups. *n* = 3, **p < 0.01.

Three binding sites between motif of ARNT and PPARα promoter were predicted **(** Figures 5A,B). In addition, ChIP results showed that ARNT directly bound to PPARα promoter (containing E1 but not E2 or E3), and luciferase assays proved that E1 (GTACGTGA) was a functional site (Figures 5C,D). Furthermore, ARNT down-regulation markedly upregulated the level of PPARα and downregulated the expression of CD36, while knockdown PPARα reversed these phenomena (Figure 5E). All these results indicated that ARNT could regulate the expression of PPARα via binding to its promoter.

### PPARα Reversed MALAT1 Knockdown-Induced Inhibition of Lipid Accumulation in FFA-Treated Hepatocytes

**FIGURE 6:**
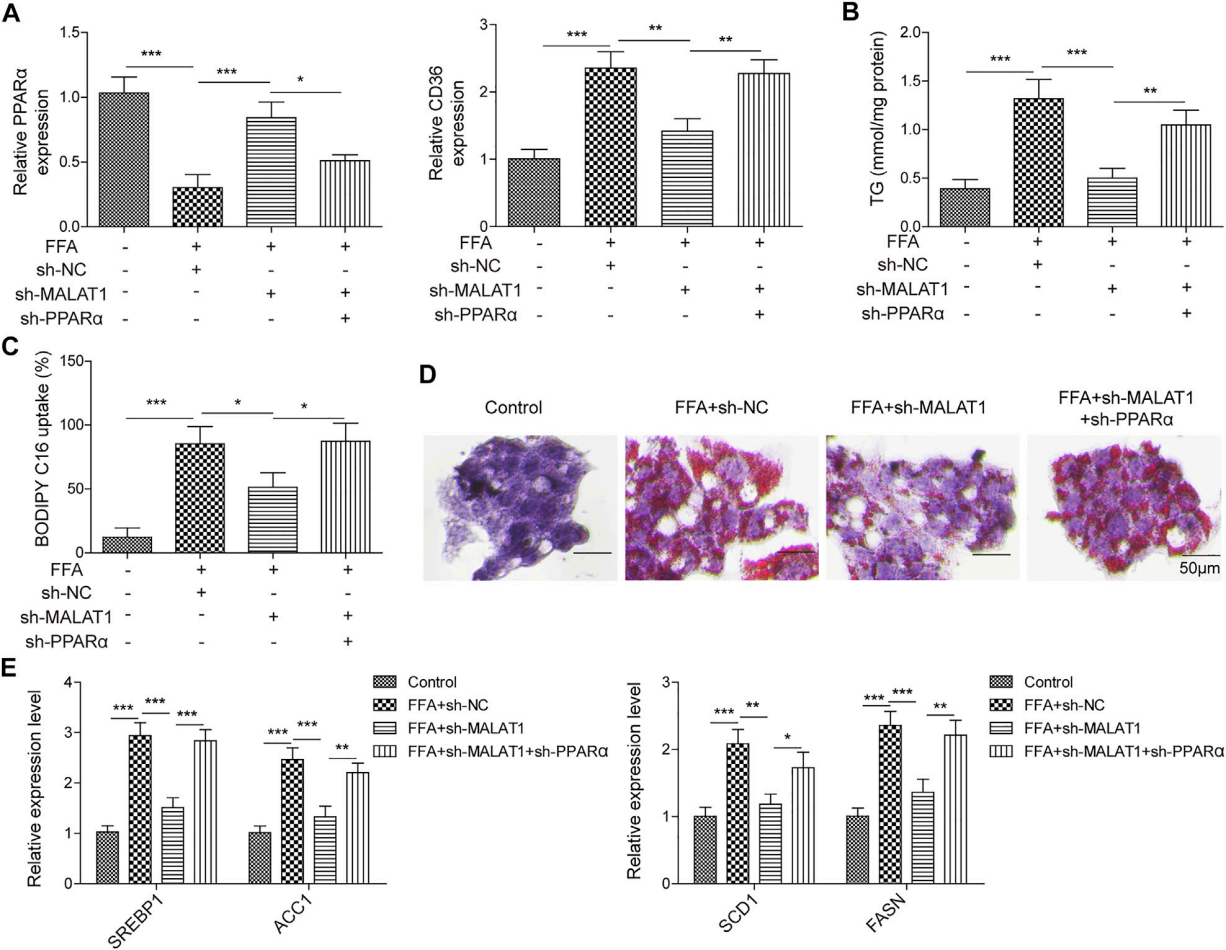
PPARα reversed the influence of MALAT1 on lipid accumulation induced by FFA in hepatocytes. **(A)** PPARα and CD36 expression were examined by qPCR. **(B)** TG level was detected by the TG assay kit. **(C)** Bodipy-labeled fatty acid uptake assay was used for assessment of fatty acid uptake. **(D)** Lipid accumulation was detected by Oil Red O staining. **(E)** SREBP1, ACC1, SCD1 and FASN levels were detected by qPCR. Data were presented as the mean ± SD. One-way analysis of variance was used among multiple groups. *n* = 3, *p < 0.05, **p < 0.01.

Finally, we investigated the detailed relation between PPARα and MALAT1 in lipid accumulation. As shown in Figure 6A, MALAT1 knockdown significantly upregulated the expression of PPARα and downregulated the level of CD36, and sh-PPARα significantly reversed the effects of sh-MALAT1. In addition, knockdown of PPARα also reversed sh-MALAT1-decreased TG level in FFA-induced cells (Figure 6B). The same trend could be found in BODIPY-labeled fatty acid uptake and Oil Red O staining assays (Figures 6C,D), indicating that PPARα reversed the effect of MALAT1 shRNA on FFA-induced lipid accumulation. Furthermore, this conclusion could be further confirmed by the qPCR, which showed that PPARα blocked MALAT1-induced downregulation of SREBP1, ACC1, SCD1 and FASN level in FFA-treated HepG2 cells (Figure 6E). Collectively, MALAT1 may modulate FFA-induced hepatic lipid accumulation through miR-206/ARNT axis.

### Knockdown of MALAT1 Alleviated the Symptom of NAFLD *in Vivo*

**FIGURE 7:**
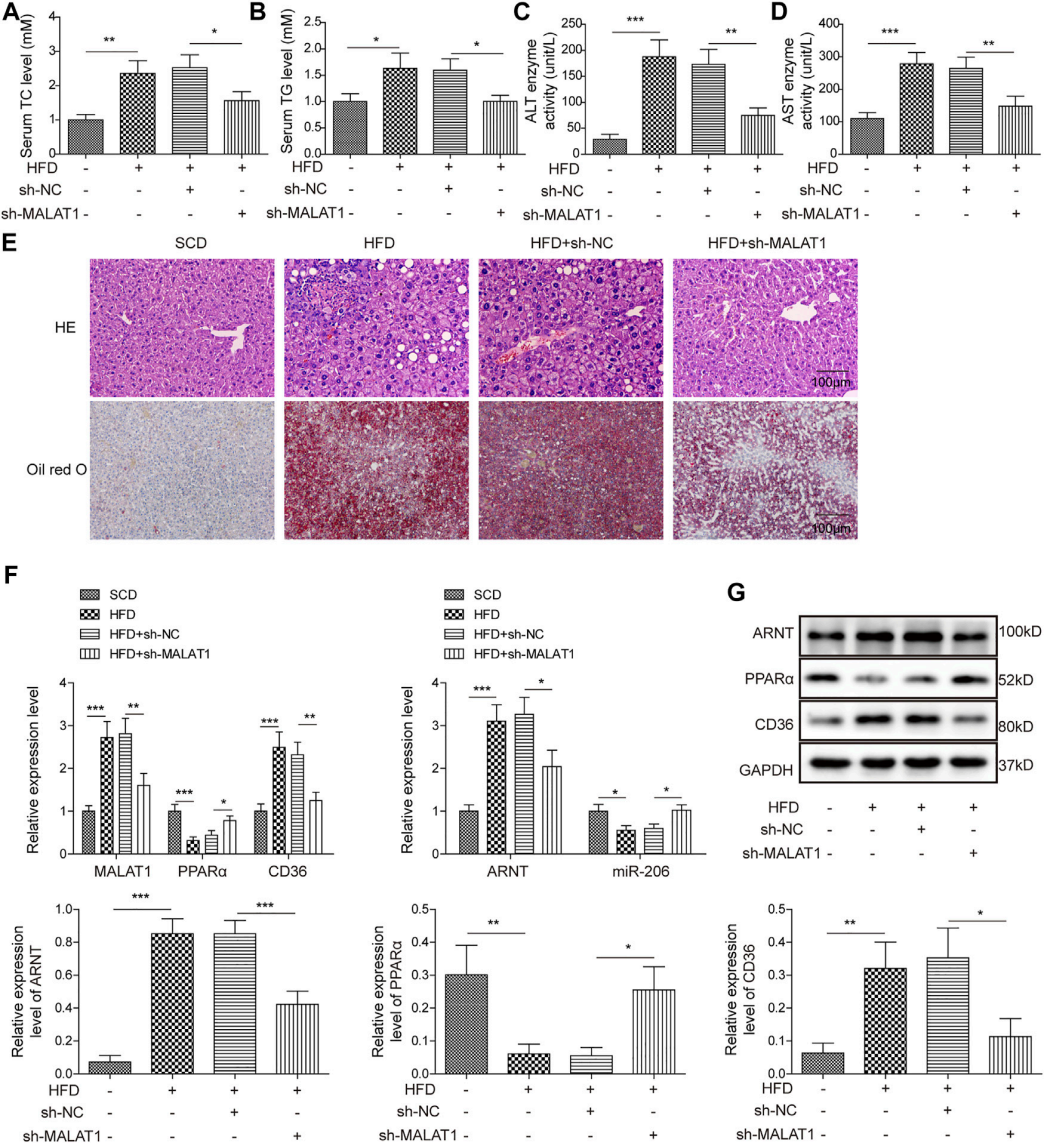
Knockdown of MALAT1 alleviates NAFLD in mice. **(A)** The level of TC in mice was detected by TC assay kit. **(B)** The level of TG in mice was detected by TG assay kit. **(C)** The ALT activity was investigated by ALT kit. **(D)** The AST activity was investigated by AST kit. **(E)** The histological changes and lipid accumulation in liver tissues of mice were detected by H&E and Oil red O staining, respectively. **(F)** The levels of MALAT1, miR-206, ARNT, PPARα and CD36 in tissues of mice were detected by qPCR. **(G)** The protein levels of ARNT, PPARα and CD36 in tissues of mice were detected by western blot. The relative expressions were quantified by normalizing to GAPDH. Data were presented as the mean ± SD. One-way analysis of variance was used among multiple groups. *n* = 6, *p < 0.05, **p < 0.01, *** <0.001.

As shown in Figures 7A,B, HFD-induced increase of TC and TG contents in mice was notably reversed by knockdown of MALAT1. Consistently, MALAT1 silencing notably rescued the effect of HFD on ALT and AST activity in mice (Figures 7C,D). Moreover, HFD significantly induced the lipid accumulation and inflammatory injury in liver tissues, which was markedly alleviated in the presence of MALAT1 shRNA (Figure 7E). In addition, the expressions of MALAT1, ARNT and CD36 in tissues of mice were notably up-regulated by HFD, while HFD exerted the opposite effect on miR-206 and PPARα levels (Figures 7F,G). Meanwhile, the effect of HFD on MALAT1, ARNT, CD36, miR-206 and PPARα levels was significantly abolished by MALAT1 knockdown (Figures 7F,G). In summary, knockdown of MALAT1 alleviated the progression of NAFLD *in vivo*.

## Discussion

Progression of NAFLD can lead to huge healthcare concern such as advanced liver disease and insulin resistance (Watt et al., 2019). The role of lncRNA in disease has been widely focused by many scientists. There is no doubt that lncRNAs play a key regulatory role in the pathogenesis of NAFLD (Hanson et al., 2018; Zhao et al., 2018). In general, lncRNAs play two main roles: 1) binding with miRNAs and functioning as spongers to regulate the expression of downstream target genes of miRNAs; 2) binding with RNA-binding proteins to regulate downstream mechanisms (Xu et al., 2019; Ye et al., 2020). In this study, we found that MALAT1 is upregulated in NAFLD. Further experiments showed that MALAT1 could negatively regulate miR-206 by acting as a sponge, and interfere with hepatic lipid synthesis through PPARα/CD36 mediated by ARNT.

In fact, lncRNA MALAT1 has been widely studied in a variety of diseases, mainly playing a role in promoting proliferation and inflammation (Malakar et al., 2019; Cai et al., 2020). However, there are few studies on NAFLD. Recently, Li et al. Found that MALAT1 may regulate liver inflammation by activating NLRP3 inflammatory complex in chronic hepatitis B with nonalcoholic fatty liver disease (Li et al., 2021). Another study on nonalcoholic steatohepatitis fibrosis showed that MALAT1 plays a key role in liver fibrosis by regulating the chemokine CXCL5 (Leti et al., 2017). However, none of these studies involved the role of MALAT1 in hepatic lipid synthesis. In fact, the existing evidence supports that MALAT1 can regulate the process of lipid synthesis (Ebrahimi et al., 2020; Wang et al., 2021a), but the specific regulatory mechanism is still lacking. In this study, MALAT1 expression was significantly up-regulated in liver tissues of patients with NAFLD. Similarly, up-regulation of MALAT1 expression was also detected in the FFA-induced HepG2. Furthermore, fat synthesis was significantly inhibited after knockdown of MALAT1 in HepG2. Additionally, the expression of lipid synthesis related factors, SREBP1, ACC1, SCD1 and FASN was significantly down-regulated after knockdown of MALAT1, and the expression of PPARγ was also significantly down-regulated.

Studies have confirmed that miR-206 plays a key role in lipid synthesis. Wang et al. recently found that miR-206 regulates lipid synthesis and cell proliferation of hepatocellular carcinoma cells by targeting G6PD (Wang et al., 2021b). Chen et al. Found that miR-206 plays a role in inhibiting hepatic lipid synthesis in non-alcoholic fatty liver disease (Chen et al., 2021). In short, miR-206 was effective in inhibiting lipid and glucose production in the liver (Wu et al., 2017). In this study, miR-206 inhibitor reversed the lipid accumulation induced by FFA, and also reversed the inhibition of lipid synthesis induced by MALAT1 knockdown. The potential role of miR-206 in inhibiting lipid synthesis in the liver is suggested.

In the related experiments on miR-206, it was found that the expression level of ARNT was up-regulated by miR-206 inhibitor. The results suggest that ARNT may be the target gene of miR-206. However, there is no research to support this result. As a transcription factor, ARNT is widely involved in the metabolism of energy substances, including sugar metabolism and lipid synthesis. In islet beta cells, knockdown of ARNT leads to inhibition of glycolysis and suppression of lipid synthesis (Pillai et al., 2011). It is suggested that ARNT can promote lipid synthesis. But at present, the role of ARNT is divided. Research from Christopher Scott, for example, suggests that increasing ARNT expression may be a potential therapeutic approach in the transition from nonalcoholic fatty liver disease to steatohepatitis (Scott et al., 2019). This study supports that ARNT expression promotes the development of NAFLD, but further exploration is needed.

ARNT, also known as HIF-1β, forming a complex with HIF-1α participates in the emergency response under hypoxic conditions (Smythies et al., 2019). In general, ARNT expression is located in the nucleus, and it forms dimers with HIF-1α under hypoxic conditions, to regulate physiological processes such as angiogenesis and energy metabolism as transcription factor (Lee et al., 2021). Hoang et al. showed that ARNT was expressed in islet beta cells. Mice knockout for ARNT show defects in lipid metabolism (Hoang et al., 2019). In this study, the potential binding sites between ARNT and PPARα promoter were predicted by bioinformatics, and further experiments showed that ARNT could negatively regulate the transcription of PPARα and mediate expression of PPARα. PPARα, a key regulator that inhibits lipid accumulation in the liver, can further cause inhibition of CD36 expression (Bougarne et al., 2018).

CD36 is a membrane-localized protein (Li et al., 2020), and The main function of CD36 as a lipid sensor is to perform energy homeostasis, including the involvement in lipid metabolism (Martin et al., 2011). It has been suggested that CD36 can form a metabolic pathway through PPARγ and regulate lipid metabolism (Maréchal et al., 2018). Previous studies have shown that CD36 is involved in catalyzing the initial step of triglyceride hydrolysis in lipid droplets in liver cells. Knockdown of CD36 resulted in upregulation of PPARα expression and inhibition of lipid accumulation (Pu et al., 2021). In the present study, the regulatory effect on PPARα mediated by ARNT could also cause alterations in CD36 expression. These suggested a key role of CD36 in relation to the PPARs family in lipid synthesis. The results of this study also found that the MALAT1-mediated miR-206/ARNT axis participated in the regulation of CD36 expression. However, the specific mechanism between PPARα and CD36 was not addressed in this study. Meanwhile, no referable evidence was found at present. It is worth spending efforts in future studies.

Recent studies reported that lncRNAs were involved in NAFLD development. For instance, Ye et al. found that knockdown of lncRNA NEAT1 alleviated the progression of NAFLD via mediation of miR-129-5p/SOCS2 signaling pathway (Ye et al., 2020). Wang et al. reported that lncRNA H19 accelerated hepatic lipogenesis in NAFLD through up-regulating both mTORC1 signalling axis and MLXIPL transcriptional network (Wang et al., 2020). Additionally, MALAT1 was up-regulated in liver tissues of patients with NAFLD (Greco et al., 2008; Hanson et al., 2018). Thus, our research was in consistence with the previous studies. Notably, FFA-induced lipid accumulation in hepatocytes can be largely reversed by the knockdown of MALAT1.

*Via* miRNA response elements (MREs), lncRNA could competitively combine miRNA to reduce its ability which influenced its target genes. The relationship between lncRNA and miRNA was called competitive endogenous RNA (ceRNA) network. MiRNA consists of about 22 nucleotides without coding function (Zhang et al., 2017), including miR-206 (Wu et al., 2017), miR-30b (Dai et al., 2019), and miR-130a (Wang et al., 2017), etc. Recently, it has been reported that miR-130 could act as a key regulator in NAFLD (Kim et al., 2017). Besides, miR-206 was reported to prevent both NAFLD and hyperglycemia progression (Zhong et al., 2013). Wu et al. demonstrated that miR-206 could both inhibit Srebp1c-induced lipogenesis and activate insulin signaling pathway (Wu et al., 2017). In this study, MALAT1 could activate ARNT through sponging miR-206. In addition, miR-206 down-regulation could reverse the effect of MALAT1 knockdown on FFA-induced lipid accumulation in hepatocytes.

Furthermore, PPARα and CD36 were essential mediators in fat metabolism and they participated in the process of NAFLD (Motojima et al., 1998). Here, the present study revealed the synergistically regulatory effect of ARNT and PPARα as follows: ARNT could inhibit the expression of PPARα through binding with its promoter. Moreover, knockdown of MALAT1 significantly upregulated the expression of PPARα and downregulated the level of CD36, and these phenomena were rescued by knockdown of PPARα.

There are some limitations in this study as follows: 1) more miRNAs targeted by MALAT1 remain to be further explored; 2) more rescue experiments should be performed to verify the relation among MALAT1, miR-206 and ARNT. Therefore, more investigations are needed in future.

In conclusion, this study revealed that MALAT1 could promote hepatic lipogenesis in nonalcoholic fatty liver disease by modulating miR-206/ARNT axis. Thereby, our research might provide new ideas for developing new strategies against NAFLD.

## New and Noteworthy

MALAT1 knockdown reversed FFA-induced lipid accumulation in hepatocytes. The effect of MALAT1 knockdown on lipid accumulation could be restored by miR-206 inhibitor. MALAT1 promoted ARNT expression through binding with miR-206. ARNT inhibited the expression of PPARα binding with PPARα promoter.

## Data Availability

The original contributions presented in the study are included in the article/supplementary material, further inquiries can be directed to the corresponding author.
